# Prognostic Value of a Novel Parameter in Patients with Infective Endocarditis

**DOI:** 10.1155/2022/1042780

**Published:** 2022-04-13

**Authors:** Ying Chen, Jingping Liu, Tengfei Qiao, Mengxiao Xie, Zhenzhen Cai, Jun Zhou

**Affiliations:** ^1^Department of Laboratory Medicine, The First Affiliated Hospital of Nanjing Medical University, Nanjing, Jiangsu, China; ^2^Branch of National Clinical Research Center for Laboratory Medicine, Nanjing, Jiangsu, China; ^3^Department of Laboratory Medicine, Nanjing Lishui District Hospital of Traditional Chinese Medicine, Nanjing, Jiangsu, China

## Abstract

**Background:**

Infective endocarditis (IE) has a high rate of mortality and the prognosis of IE was poor. The purpose of this investigation was to explore the value of lactate dehydrogenase (LDH)/lymphocyte and compare it with LDH/lymphocyte percentage (L-LWR) in predicting the in-hospital mortality in IE patients.

**Methods:**

The investigation cohort contained 147 IE patients between January 2017 and December 2019. We retrospectively went over the medical records and selected admission indexes.

**Results:**

Compared with IE patients with adverse events, significantly higher levels of LDH/lymphocyte and significantly lower levels of L-LWR were discovered in IE patients without adverse events. After adjustments, L-LWR (odds ratio (OR): 4.558, 95% confidence interval (CI) 1.362-15.256, *P* = 0.014) still maintained its significant independence. In addition, L-LWR had the highest area under curve (AUC) (0.780, 0.704-0.844, *P* < 0.001) with good sensitivity (81.89%) and specificity (65.00%) when 34 was selected as the best cutoff value.

**Conclusions:**

L-LWR is a reliable, low-priced, easily applicable, and independent prognostic parameter for in-hospital death with good performance in patients with IE.

## 1. Introduction

Infective endocarditis (IE), as one of the infectious diseases, has a high rate of mortality [1]. Looking back at the past, the etiology and epidemiology of IE have altered [2]. However, with the development of the diagnostic and therapy tools, the prognosis of IE has not been significantly improved. The clinical manifestations of IE is affected by varying factors, including advanced age, underlying disease, and complications [3]. Thus, timely and accurate diagnosis, prognosis, and administration of IE patients are significant.

The lymphocyte percentage (LWR) was an effective prognostic tool for patients with colorectal cancer [4]. Lactate dehydrogenase/lymphocyte (LDH/lymphocyte) is associated with mortality in COVID-19 patients [5, 6]. However, there is no study that assessed the role of LDH/lymphocyte or LDH/LWR (named as L-LWR) in IE patients. Therefore, we conducted the work to explore the value of LDH/lymphocyte and L-LWR and compare them in predicting the in-hospital mortality in IE patients.

## 2. Materials and Methods

### 2.1. Study Population

The investigation cohort contained 147 IE patients (confirmed by the modified Duke criteria [7]) who were admitted to the hospital between January 2017 and December 2019. We retrospectively went over the medical records and selected these indexes including blood culture results, surgical treatment (during the hospital), demographic characteristics, microbiological parameters, blood routine parameters, transaminase, alkaline phosphatase (ALP), glutamyl transpeptidase (GGT), LDH, creatine kinase (CK), kidney function, and echocardiographic data at admission. Short-term outcomes were acquired from the telephone call or the electronic medical records. This study was approved by local hospital and in consistency with the Declaration of Helsinki.

### 2.2. Statistical Analysis

Quantitative variables are reported as mean ± standard deviation (SD). On univariate analysis, Student's *t*-tests were used for continuous variables and chi-square tests were used for categorical variables. *P* values less than 0.05 were considered to be statistically significant. Clinical risk factors affecting in-hospital death were determined on multiple analysis. All analyses were performed using SPSS version 21.0 (IBM Co., Armonk, NY, USA).

## 3. Results

### 3.1. Patients' Characteristics

A total of 147 IE patients were entered in the clinical investigation after exclusion. The characteristics of the included IE patients were displayed according to the short-term outcomes. The short-term death rate of the cohort was 13.6% (*n* = 20). Overall, male make up the majority (69.4%). Compared with IE patients with adverse events (in-hospital death), significantly higher levels of LWR and LDH/lymphocyte (0.17 ± 0.08 vs. 0.10 ± 0.06, *P* = 0.001; 416.98 (56.06, 2340.00) vs. 232.85 (65.19, 3092.68), *P* = 0.001), significantly lower levels of L-LWR (39.90 (11.81, 418.92) vs. 20.71 (3.51, 265.91), *P* < 0.001), age, WBC, neutrophil, LDH, CK, urea nitrogen (UREA), creatinine (CREA) and surgery were found in IE patients without adverse events. No difference in gender, lymphocyte, hemoglobin (HB), platelets, transaminase, ALP and GGT were discovered in IE patients with and without adverse events (Table 1).

In addition, blood culture details were viewed in Table 1. The results suggested that streptococci (*n* = 30 cases, 20.4%) and staphylococcus (*n* = 12 cases, 8.2%) were the major pathogen.

### 3.2. Association of L-LWR Levels with In-Hospital Death in IE Patients

In-hospital mortality (adverse event) was named as all-cause death (within 30 days). Patients with elevated L-LWR levels were at high risk in in-hospital mortality. To assess whether L-LWR is a prognosis factor for in-hospital mortality. We first classify indicators less than 0.05 in Table 1 into categorical variables and then put these indicators into the model for multivariate analysis. After adjustments, age (odds ratio (OR): 11.334, 95% confidence interval (CI) 1.035-124.067, *P* = 0.047), surgery (OR: 4.137, 95% CI 1.231-13.902, *P* = 0.022), CK (OR: 4.231, 95% CI 1.215-14.735, *P* = 0.023), UREA (OR: 3.417, 95% CI 1.020-11.443, *P* = 0.046), and L-LWR (OR: 4.558, 95% CI 1.362-15.256, *P* = 0.014) still maintained their significant independence (Table 2).

**Figure 1:**
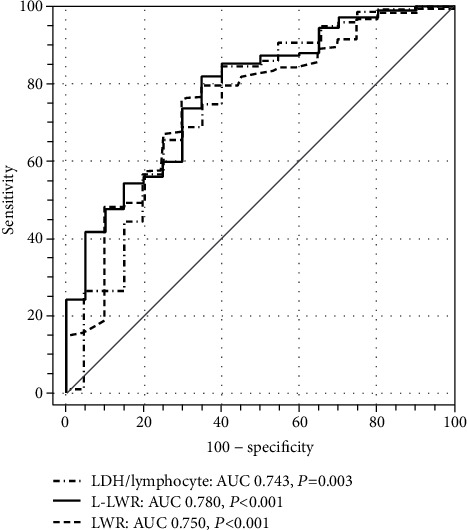
ROC curves for 30-day mortality.

Receiver operating characteristic (ROC) analysis was recommend to check the area under the curve (AUC) for each independent factors. L-LWR had the highest AUC (0.780, 0.704-0.844, *P* < 0.001) with good sensitivity (81.89%) and specificity (65.00%) when 34 was selected as the best cutoff value (Figure 1).

## 4. Discussion

In recent times, investigators have made a great effort to explore factors to judge the prognosis of patients with different diseases, such as IE [8–12]. LDH, lymphocyte, and LWR are inexpensive, easy to get, and automated factors that timely and effectively forecast the prognosis of the patients with different sicknesses [13–17]. Previous researches suggested that the performance (accuracy/precision) of the combination of LDH and lymphocyte (LDH/lymphocyte) was better than that of LDH and lymphocyte alone [5]. However, regarding the value of LDH/lymphocyte and L-LWR in the prognosis of patients with IE, it is still blank. Therefore, our intent is to question the association between the combination (LDH/lymphocyte (L-LWR)) and short-term outcomes. In this research, L-LWR was firstly verified as an independent in-hospital mortality index: IE patients with elevated L-LWR have worse prognosis than those with low- L-LWR. Furthermore, compared with a single biomarker (LDH and lymphocyte) and ratio (LWR and LDH/lymphocyte), L-LWR, based on LDH, lymphocyte, and WBC, was the best combination with outstanding prognosis.

LDH, a tetrameric enzyme, could catalyze pyruvic acid [18]. An elevation of LDH is often accompanied by organ damage [19]. Therefore, we speculate that LDH may play a role in predicting adverse events. To patients with tumor, such as small cell lung cancer, hepatocellular carcinoma, and acute pancreatitis, elevated LDH represents a bad prognosis [15, 20, 21]. These discoveries confirmed our speculation.

Lymphocytes, as a simple and cheap biomarker, have been widely investigated. Neuroblastoma patients with high monocyte × lymphocyte indicate a good prognosis [22]. While to HER-2-positive breast cancer patients treated with trastuzumab, an elevated lymphocyte is significantly related to a bad prognosis [23]. Further, compared with lymphocyte, LWR is a better predictor to forecast the prognosis of advanced cancer patients with palliative care [24]. In this research, we also found that LWR could be used to predict the adverse events.

Overall, LDH, lymphocyte, LWR, and LDH/lymphocyte play an important role in predicting the adverse events. So far, there is no research implicating these prognostic factors in a study and that makes a comparison. In this study, we recommend a new parameter (L-LWR) and compare it with the abovementioned indicators. The results pointed out that L-LWR was not only an independent prognosis factor but also an effective and the best prognostic index for IE patients.

The retrospective research had some limitations. Firstly, selection bias exists owning to a single-center investigation. Secondly, we did not assess the value of L-LWR in predicting long-term outcomes. Thirdly, the potential mechanisms have not been explored. Large sample size and multicenter work should be conducted in the future.

## 5. Conclusion

L-LWR is a reliable, low-priced, easily applicable, and independent prognostic parameter for in-hospital death with good performance in patients with IE.

## Figures and Tables

**Table 1:** Clinical characteristics of the study population.

Variable	Nonsurvivor group (*n* = 20)	Survivor group (*n* = 127)	*P* value
Age (years)	57 ± 11.74	50 ± 14.63	0.044
Gender (male, *n*%)	16 (80.0%)	86 (67.7%)	0.271
WBC (×10^9^/L)	12.68 ± 5.79	9.28 ± 4.61	0.004
Lymphocyte (×10^9^/L)	1.12 ± 0.93	1.36 ± 0.61	0.131
Neutrophil (×10^9^/L)	10.60 ± 5.66	7.25 ± 4.31	0.002
LWR	0.10 ± 0.06	0.17 ± 0.08	0.001
HB (g/L)	102.70 ± 13.75	106.62 ± 20.71	0.415
PLT (×10^9^/L)	175.40 ± 105.34	202.01 ± 111.37	0.319
ALT	22.7 (3.9, 2433.3)	24.1 (3.0, 280.2)	0.468
AST	27.5 (13.7, 3654.2)	24.2 (9.1, 218.6)	0.360
ALP	136.7 ± 95.10	108.21 ± 55.26	0.058
GGT	42.9 (13.90, 216.40)	46.3 (11.10, 598.20)	0.769
LDH	332 (212.00, 2536.00)	292 (128.00, 644.00)	0.010
CK	48 (13.00, 742.00)	28 (6.00, 523.00)	0.002
UREA	10.00 ± 6.23	6.45 ± 4.43	0.002
CREA	83.8 (50.7, 902.0)	69.7 (37.1, 944.2)	0.028
LDH/LY	232.85 (65.19, 3092.68)	416.98 (56.06, 2340.00)	0.001
L-LWR	39.90 (11.81, 418.92)	20.71 (3.51, 265.91)	<0.001
Etiology			
Staphylococcus, *n* (%)	1 (5.00%)	11 (8.67%)	0.581
Streptococcus, *n* (%)	6 (30.00%)	24 (18.90%)	0.255
Others, *n* (%)	1 (5.00%)	8 (6.30%)	0.823
Culture negative, *n* (%)	12 (60.00%)	84 (66.14%)	0.595
Surgery (%)	40.00%	81.11%	<0.001

ALP: alkaline phosphatase; ALT: alanine aminotransferase; AST: aspartate aminotransferase; CK: creatine kinase; CREA: creatinine; GGT: glutamyl transpeptidase; HB: hemoglobin; LDH: lactate dehydrogenase; LWR: lymphocyte-to-white blood cell ratio; L-LWR: LDH-to LWR; PLT: platelet; UREA: urea nitrogen; WBC: white blood cell.

**Table 2:** Multivariable logistic regression of in-hospital mortality for patients with infective endocarditis.

Variables	Univariate analysis			Multivariate analysis			Forest plot
	HR	95% CI	*P* value	HR	95% CI	*P* value	
Age	10.417	1.351-80.469	0.004	11.334	1.035-124.067	0.047	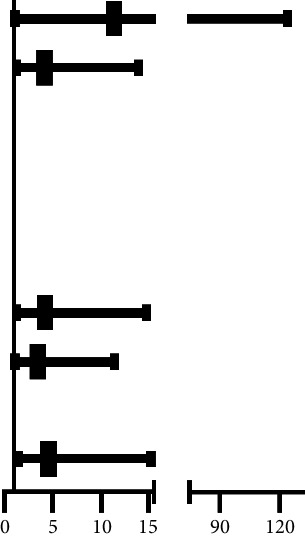
Surgery	6.438	2.371-17.478	<0.001	4.137	1.231-13.902	0.022	
WBC	4.103	1.544-10.900	0.008				
Neutrophil	4.645	1.740-12.401	0.003				
LWR	6.005	2.196-16.420	<0.001				
LDH	5.654	1.257-25.438	0.012				
CK	5.467	1.865-16.025	0.001	4.231	1.215-14.735	0.023	
UREA	7.571	2.759-20.782	<0.001	3.417	1.020-11.443	0.046	
CREA	3.785	1.435-9.980	0.008				
L-LWR	8.398	3.017-23.377	<0.001	4.558	1.362-15.256	0.014	

CI: confidence interval; CK: creatine kinase; CREA: creatinine; HR: hazard ratio; LDH: lactate dehydrogenase; LWR: lymphocyte-to-white blood cell ratio; L-LWR: LDH-to LWR; UREA: urea nitrogen; WBC: white blood cell.

## Data Availability

The datasets are available from the corresponding author upon reasonable request.
